# Roles of ncRNAs as ceRNAs in Gastric Cancer

**DOI:** 10.3390/genes12071036

**Published:** 2021-07-02

**Authors:** Junhong Ye, Jifu Li, Ping Zhao

**Affiliations:** 1 State Key Laboratory of Silkworm Genome Biology, Biological Science Research Center, Southwest University, Chongqing 400716, China; yejunhong0129@outlook.com; 2 College of Sericulture, Textile and Biomass Sciences, Southwest University, Chongqing 400716, China; lijifu0103@163.com

**Keywords:** non-coding RNAs, competitive endogenous RNA, gastric cancer

## Abstract

Although ignored in the past, with the recent deepening of research, significant progress has been made in the field of non-coding RNAs (ncRNAs). Accumulating evidence has revealed that microRNA (miRNA) response elements regulate RNA. Long ncRNAs, circular RNAs, pseudogenes, miRNAs, and messenger RNAs (mRNAs) form a competitive endogenous RNA (ceRNA) network that plays an essential role in cancer and cardiovascular, neurodegenerative, and autoimmune diseases. Gastric cancer (GC) is one of the most common cancers, with a high degree of malignancy. Considerable progress has been made in understanding the molecular mechanism and treatment of GC, but GC’s mortality rate is still high. Studies have shown a complex ceRNA crosstalk mechanism in GC. lncRNAs, circRNAs, and pseudogenes can interact with miRNAs to affect mRNA transcription. The study of the involvement of ceRNA in GC could improve our understanding of GC and lead to the identification of potential effective therapeutic targets. The research strategy for ceRNA is mainly to screen the different miRNAs, lncRNAs, circRNAs, pseudogenes, and mRNAs in each sample through microarray or sequencing technology, predict the ceRNA regulatory network, and, finally, conduct functional research on ceRNA. In this review, we briefly discuss the proposal and development of the ceRNA hypothesis and the biological function and principle of ceRNAs in GC, and briefly introduce the role of ncRNAs in the GC’s ceRNA network.

## 1. Introduction

A non-coding RNA (ncRNA) is a type of RNA that does not have the function of a coding protein [1]. NcRNAs, which account for 98% of the human genome, include ribosomal RNAs (rRNAs), short ncRNAs, circRNAs, pseudogenes, and many lncRNAs [2]. For a long time, lncRNAs, circRNAs, and pseudogenes were regarded as useless components in the genome. In 1976, scholars discovered the existence of circRNA (pathogenic single-stranded circular virus) in higher plants [3]. In 1977, the first pseudogene was discovered in the *Xenopus* genome [4]. In the 1990s, researchers discovered an imprinted gene, lncRNA *H19*, which forms the *H19/IGF-2* imprinted gene group with the similarly-located insulin-like growth factor 2 [5]. At the same time, other studies showed that the lncRNA *XIST* can participate in the transcriptional regulation of genes on sex chromosomes [6,7]. Thus, ncRNA began receiving attention. *HOTAIR*, another lncRNA, was discovered in 2007. Studies have shown that it can enhance the PRC2 activity of the *HOXD* locus and participate in PRC2-mediated chromatin silencing [8]. In 2013, a study revealed, for the first time, that circRNA could be used as a miRNA sponge to adsorb miRNA, thereby affecting gene expression [9]. With the deepening of research, it was found that lncRNAs, circRNAs, and pseudogenes can play biological functions in immune response [10], nerve conduction [11], growth and development [11], and stress response [12]. With the help of microarray and RNA sequencing technology, people have ascertained that lncRNAs, circRNAs, and pseudogenes are involved in regulating various tumor cell biological activities [13].

It was discovered that ncRNAs contain miRNA response elements (MREs) and act as a miRNA sponge, and an increasing number of studies have shown that they participate in the formation of a complex regulatory network. The ceRNA hypothesis proposes that certain transcripts, such as lncRNAs, circRNAs, pseudogenes, and mRNAs, have MREs in common, regulating the transcription of gene expression through competitive binding of miRNAs [14]. Thus, they are each other’s ceRNA. It has been 10 years since the ceRNA hypothesis was put forward, and research on ceRNA has been steadily increasing yearly. Researchers found that the ceRNA network plays an important role in cardiovascular diseases such as myocardial hypertrophy [15,16], myocardial infarction [17,18], atherosclerosis [19,20,21,22], neurodegenerative diseases such as Alzheimer’s disease [23,24], Parkinson’s disease [25,26], Huntington’s disease [27,28], and neuroimmune diseases such as progressive muscular dystrophy and cocaine syndrome [29,30,31]. Therefore, studying the ceRNA regulatory network is of great significance in understanding the diseases’ occurrence and development, and improving clinical diagnosis, treatment methods, and prognosis.

Cancer became the main cause of death and the single most important obstacle to increasing people’s life expectancy in the 21st century. Cancer is mainly related to genetic factors [32], immune factors [33], endocrine factors and other endogenous factors, as well as living habits [34,35], environmental pollution [36], biological factors [37], and other exogenous factors. ceRNAs play an important role in cancer progression, including gastric cancer (GC), colon cancer, liver cancer, breast cancer, and lung cancer [38,39]. GC is a common cancer worldwide. Studies have found that lncRNAs, circRNAs, and pseudogenes such as ceRNAs can participate in biological behaviors such as GC proliferation, differentiation, and cell resistance. Therefore, an increasing number of studies on the ceRNA network in GC are expected to provide new ideas for understanding the mechanism of GC occurrence and development and simultaneously provide direction for finding new targets for treating GC.

## 2. Gastric Cancer

As the fifth-most-common cancer and the third-leading cause of cancer death worldwide, GC is a deadly digestive system disease afflicting many people. GC was responsible for over 1,000,000 new cases in 2018 and an estimated 783,000 deaths (equating to one in every 12 deaths globally) [38,40].

Global cancer statistics 2018 show that GC incidence and mortality in Asia rank first by world region. Factors that cause this disease include *Helicobacter pylori* infection, age, high salt intake, and low fruit and vegetable diets. Alcohol consumption and active tobacco smoking are also established risk factors [38].

However, the gold standard for GC diagnosis is endoscopic biopsy plus enhanced computed tomography. Many patients resist examination due to the insidious onset, unobtrusive symptoms, and invasive examination methods. Furthermore, since early GC has nonspecific symptoms, most GC patients are diagnosed at advanced stages, and the 5-year survival rates range between 20% and 30% [41,42].

Surgical treatment plus chemotherapy remains the first-line approach to provide a cure for GC. Despite advances in surgical techniques, radiotherapy, chemotherapy, and neoadjuvant therapy, chemotherapy resistance or drug resistance is still an important issue that needs to be faced because cancer cells will form a mechanism to counteract the effects of chemotherapy drugs, leading to more clones and aggressiveness, and eventually a poor prognosis. Chemoresistance can be inherent and acquired, and it is a multi-factor event, including dysregulation of key signaling pathways, acquired mutations, and DNA damage responses [43].

Therefore, exploring the pathogenesis and looking for key factors to guide diagnosis and treatment has always been a research focus.

The occurrence and development of GC is a multi-stage and multi-factor process, and its pathogenesis is complex. The current research shows that its occurrence is often related to abnormal transcription. This abnormality is not limited to abnormal protein-coding RNA (mRNA) levels and includes abnormalities in the regulatory ability of ncRNA in the genome. Studies have shown that the cancer stem cell (CSC) is one of the main reasons for the failure of cancer treatment. The expression of miRNAs plays an important role in the maintenance of stem/progenitor cells. The dysregulation of miRNAs in gastric cancer stem cells (GCSCs) is closely related to the occurrence and development of gastric cancer [44].

## 3. ceRNAs

In 2007, Ebert et al. artificially synthesized miRNA inhibitors called miRNA sponges. With an increasing number of experimental verifications and the discovery of endogenous miRNA sponges, in 2011, Salmena et al. proposed the ceRNA hypothesis for the first time. It was expounded that in addition to the traditional miRNA→RNA mode of action, there is also an RNA–miRNA–mRNA regulation mode [14,45,46,47].

Here, “ceRNA” does not refer to a specific RNA but to a brand-new mode of gene expression regulation, describing a mode of action of RNA. The mechanism of ceRNA is that when the ceRNA expression is silenced, mRNAs are transcribed and exported to the cytoplasm, where they are targeted by the miRNA-mediated silencing complex (miRNA–RISC), resulting in accelerated degradation, blocking of translation, and reduction of gene expression; Second, when the ceRNA expression is activated, there will be competition for miRNA targeting and binding to the RISC complex, reducing miRNA inhibition; the miRNA–RISC complex is isolated from the gene, resulting in increased gene expression.

ceRNAs use similar MREs to bind miRNAs, thereby indirectly regulating genes’ expression competitively. This competitive miRNA binding effect is also called miRNA sponge action. According to this theory, any RNA that contains MREs may be a ceRNA, its core is miRNAs, and its members include lncRNAs, cirRNAs, mRNAs, and pseudogenes. Among the RNAs that can be used as ceRNAs, those that regulate tumor progression play an important role [48,49].

Besides, there are multiple MREs on each mRNA so that each mRNA can have multiple miRNA pathways. Each miRNA has multiple ceRNAs, thus forming the last “many-to-many” ceRNA networks (ceRNETs). Compared with the miRNA regulation network, ceRNETs are more sophisticated and complex, involving more RNA molecules. When ceRNAs are abnormally expressed, they affect the expression of multiple target genes in the body and further influence cancer progression.

Research shows that ceRNAs play critical roles in the development and progression of cancers. Considering the complexity of the network of ceRNAs, this research is still in its infancy. At present, the most effective way to reveal the ceRNA function in cancer is to build ceRNETs first. A common research method is to obtain samples from different tissues, screen different miRNAs, lncRNAs, and mRNAs through microarray or sequencing technologies or the use of databases to collect information, screen differentially expressed RNAs, construct ceRNETs, extract key networks, and finally perform functional enrichment analysis and survival analysis to discover genes related to cancer development and prognosis [50,51,52].

The most commonly used databases are the Cancer Genome Atlas (TCGA) database and Gene Expression Omnibus (GEO) microarray datasets. Furthermore, researchers have also established some dedicated tools to facilitate the identification of ceRNA networks, including ceRDB, Linc2GO, starBase v2.0, lnCeDB, and Cupid. Details and resources are summarized in chronological order in Table 1. The functions of these tools are different. Researchers should choose according to their needs.

## 4. lncRNAs as ceRNAs in GC

lncRNAs are greater than 200 nucleotides in length molecules lacking obvious open reading frames, not translated into proteins, and widely transcribed in the genome of eukaryotic cells [69].

Recently, lncRNAs have become a research focus in the field of oncology. There are diverse mechanisms for lncRNAs to regulate miRNA. This article focuses on their actions as ceRNAs, where lncRNAs can play the role of endogenous "miRNA sponges" competing with mRNAs to bind the MREs of miRNAs, thereby inhibiting miRNA expression and its negative regulation of target genes, and participating in the occurrence and development of tumors, providing a new perspective for the study of tumor formation mechanisms and tumor detection methods [70,71,72,73,74].

### 4.1. HOTAIR

Using high-resolution chip analysis technology, scholars discovered a lncRNA transcribed from the *HOXC* locus in the study of 11 human fibroblasts and named it *HOTAIR* in 2007. *HOTAIR* was the first antisense transcription lncRNA to be discovered. It contains 2158 nucleotides, and its expression level in cancer tissues is higher than in normal tissues [8]. Studies have found that it functions as a ceRNA in the occurrence and development of GC, breast cancer [75], lung cancer [76], liver cancer [77], and other tumors [78,79,80], and it is also related to drug resistance [81].

In 2016, a study showed that, in GC, *HOTAIR* directly binds to miR-126 and inhibits its expression, thus enhancing the expression of VEGFA and PIK3R2 and activating the PI3K/AKT/MRP1 pathway. *HOTAIR* acts as a ceRNA to promote cisplatin resistance [82]. In 2017, scholars found that the expression of *HOTAIR* was negatively correlated with the expression of miR-34a. The up-regulation of miR-34a caused by the down-regulation of *HOTAIR* can reduce cisplatin resistance in GC. The effect of the *HOTAIR*/miR−34a axis on GC cells may be related to PI3K/Akt and Wnt/β-catenin signaling pathway [83]. In 2018, it was found that the expression of *HOTAIR* was negatively correlated with the expression of miR-217. *HOTAIR* inhibits the expression of miR-217 and promotes the expression of GPC5 and PTPN14 as a ceRNA. Overexpression of *HOTAIR* inhibited the expression of miR-217 and enhanced the resistance of GC cells to paclitaxel and adriamycin [84]. In the same year, scholars discovered that *HOTAIR* directly targets miR-17-5p, and PTEN is modified by *HOTAIR* and miR-17-5p, which affects the proliferation and apoptosis of GC cells [85]. That year a study also found that the expression of *HOTAIR* was negatively correlated with the expression of miR-454-3p. By inhibiting the activity of STAT3/cyclin D1, down-regulating *HOTAIR* to stimulate the expression of miR-454-3p could inhibit the cell growth of GC [86]. Researchers then found that *HOTAIR* and miR-126 negatively regulate each other, which can increase or decrease the expression of CXCR4. Highly expressed *HOTAIR* promotes the proliferation and metastasis of GC through the miR-126/CXCR4 axis and downstream signaling pathways [87]. In addition, miR-618 is also a direct target of *HOTAIR*. The silence of *HOTAIR* makes miR-618 spongy, thereby blocking the development of GC and inhibiting the growth of xenograft tumors in vivo [88]. In 2020, researchers discovered a negative regulatory relationship between *HOTAIR* and miR-1277-5p. *HOTAIR* regulates the growth of GC by stimulating miR-1277-5p and up-regulating COL5A1 [89]. In the same year, a study found that *HOTAIR* can promote the carcinogenesis of GC by regulating the levels of miRNA in cells and exosomes. Over-expressed *HOTAIR* induced the degradation of miR-30a or -b, thus acting as a ceRNA [90]. The latest research shows that *HOTAIR* and miR-148b can induce the methylation of the tumor suppressor gene PCKG10 and promote GC [91]. These data indicate that *HOTAIR* can promote the occurrence and development of GC in various ways and enhance the drug resistance of GC cells as a ceRNA.

### 4.2. XIST

*XIST* is located in the X chromosome’s inactive central region, affecting the activation of X-chromosome-related genes [6,7]. Studies have found that *XIST* is abnormally expressed in various tumors and acts as a ceRNA to mediate tumor cell proliferation, migration, invasion, and drug resistance [92,93].

lncRNA *XIST* is significantly up-regulated in GC tissues and cell lines, and there is a negative correlation between its expression level and that of miR-101. Down-regulating the expression of *XIST* can inhibit the occurrence, development, and metastasis of GC by regulating the expression of EZH2 through miR-101 [94]. Studies have found that *XIST* promotes cell development from the G1 phase to the S phase and protects cells from apoptosis. *XIST* participates in the miR-497/MACC1 axis to regulate the proliferation and invasion of GC cells [95]. In addition, the researchers found that the expression of *XIST* and miR-185 are negatively correlated. miR-185 can negatively regulate the expression of TGF-β1 in vitro, and *XIST* can be used as a ceRNA to participate in the development of GC through the miR-185/TGF-β1 axis [96]. In 2020, studies found that *XIST* acts as a ceRNA in GC to regulate JAK2 by competing with miR-337. Up-regulation of miR-337 can reduce the expression of JAK2, thereby inhibiting the proliferation and migration of GC cells [97]. In addition to competing with miR-337, *XIST* can up-regulate the expression of PXN by competitively binding miR-132, which can enhance the ability to form GC cell proliferation, and migration. In studying the relationship between *XIST* and cisplatin resistance in GC, researchers found that *XIST* and miR-let-7b levels are negatively correlated, and the interaction between the two promotes cisplatin resistance [98].

### 4.3. H19

As the first imprinted gene to be discovered, lncRNA *H19* is located on the *H19/IGF2* gene cluster of human chromosome 11p15 [5]. With the deepening of research, it was found that lncRNA *H19* plays an important role in the occurrence and development of cancer. It acts as an oncogene in some tumors to mediate the tumor process, while in others it plays a role as a tumor suppressor gene [99,100,101].

Studies have found that the expression of *H19* is positively correlated with the expression of miR-675. The up-regulated expression of *H19* and miR-675 can promote cell proliferation and inhibit cell apoptosis. The *H19*/miR-675 axis promotes GC’s occurrence and development through the FADD/caspase 8/caspase 3 signaling pathway [102]. In 2018, researchers found that the expression of *H19* was negatively correlated with the expression of miR-let-7c. miR-let-7c belongs to the let-7 family and functions as a tumor suppressor gene. Silencing *H19* resulted in a significant increase in let-7c expression, while HER2 protein expression decreased, indicating that *H19* competes with miR-let-7c as a ceRNA in GC and regulates HER2 expression [103]. In the analysis of the GC ceRNA network, scholars found that the differentially regulated miR-21 and miR-148a play an important role in coordinating the sponge activity of *H19*, and the overexpression of *H19* may be a landmark event in gastric tumorigenesis [104]. In 2019, studies showed that *H19* expression is inversely proportional to miR-22-3p expression in GC tissues, and the inhibition of Snail1 can partially reverse the cell growth and metastasis induced by miR-22-3p down-regulation. *H19* promotes tumor growth and metastasis through the miR-22-3p/Snail1 signaling pathway [105]. In 2020, when analyzing the lncRNA–miRNA–mRNA network of GC, scholars found that *H19*, miR-29a-3p, *COL3A1*, *COL5A2*, *COL1A2*, and *COL4A1* can form a ceRNA network. *H19* stimulates miR-29a-3p to promote GC [106]. The latest research shows that knocking down the expression of *H19* can promote the up-regulation of miR-138, and E2F2 can be negatively regulated by miR-138, thereby inhibiting the proliferation and invasion of GC, increasing the rate of apoptosis [107].

### 4.4. MALAT1

In 2003, researchers discovered a differentially expressed gene in tumor cells of patients with early-stage non-small-cell lung cancer [108]. After screening and comparison, they found that it was an alpha transcript that had been described in 1997 and is known as *MALAT1* [109]. Studies have shown that *MALAT1* is involved in tumor proliferation, metastasis, apoptosis, epigenetic regulation, cell signal transduction, and other processes [110,111,112]. Recently, *MALAT1* has attracted more researchers’ attention due to its role as a ceRNA in GC [113].

In 2016, scholars found that *MALAT1* is up-regulated in GC tissues. Knockdown of *MALAT1* can negatively regulate miR-202 and significantly reduce the expression of Gli2, thereby inhibiting the proliferation of GC cells and inducing apoptosis [114]. The expression of MALAT1 is relatively high in the cancer tissues of patients with short survival and poor prognosis. *MALAT1* can sponge *miR-1297*, and they are negatively correlated. The up-regulation of *MALAT1* leads to *miR-1297*, thus reducing the ability to inhibit the expression of HMGB2 [115]. A 2017 study showed that the expression of *MALAT1* is related to the chemoresistance of GC cells. As a ceRNA of miR-23b-3p, *MALAT1* can weaken the inhibitory effect of miR-23b-3p on ATG12, leading to the chemical induction of GC cell autophagy and chemical resistance [116]. The ceRNA network shows that the differentially regulated miR-21 and miR-148a play an important role in coordinating the sponging activity of *MALAT1* in GC [104]. In 2019, scholars found that *MALAT1* inhibits miR-30b expression as a ceRNA in the study of chemical resistance to GC. *MALAT1* enhanced autophagy-related chemical resistance of GC by inhibiting the miR-30b/ATG5 axis [117]. Research in the same year showed that *MALAT1* acts as a sponge of miR-125a, and the dysregulation of the *MALAT1*/miR-125a axis causes IL-21R to play a carcinogenic role in GC [118]. *MALAT1* can also competitively bind to miR-181a-5p, which prevents miR-181a-5p from binding to AKT3 mRNA, thereby up-regulating the level of AKT3 protein and ultimately promoting tumor growth in GC [119]. In 2020, when investigating the autophagy activity of GC tissues, researchers found that *MALAT1* can inhibit the expression of miR-204 in GC cells and prevent miR-204 from down-regulating LC3B and transient receptor potential melastatin 3 (transient receptor potential melastatin 3), which activates autophagy and promotes cell proliferation [120]. *MALAT1* is also negatively correlated with the expression of miR-22-3p. MiR-22-3p can negatively regulate ErbB3. The high expression of *MALAT1* promotes proliferation and prevents apoptosis of GC cells by down-regulating miR-22-3p and up-regulating ErbB3. In the study of *MALAT1* and miR-22-3p, it was also found that *MALAT1* regulates ZFP91 through sponge miR-22-3p to enhance GC cells’ resistance to oxaliplatin (OXA) [121]. The latest research shows that hydrogen gas can inhibit the proliferation of GC cells and the expression of *MALAT1* and EZH2, up-regulating the expression of miR-124-3p at the same time. It shows that the expression of *MALAT1* and miR-124-3p is negatively correlated. Overexpression of *MALAT1* can eliminate the effect of hydrogen [122].

**Figure 1:**
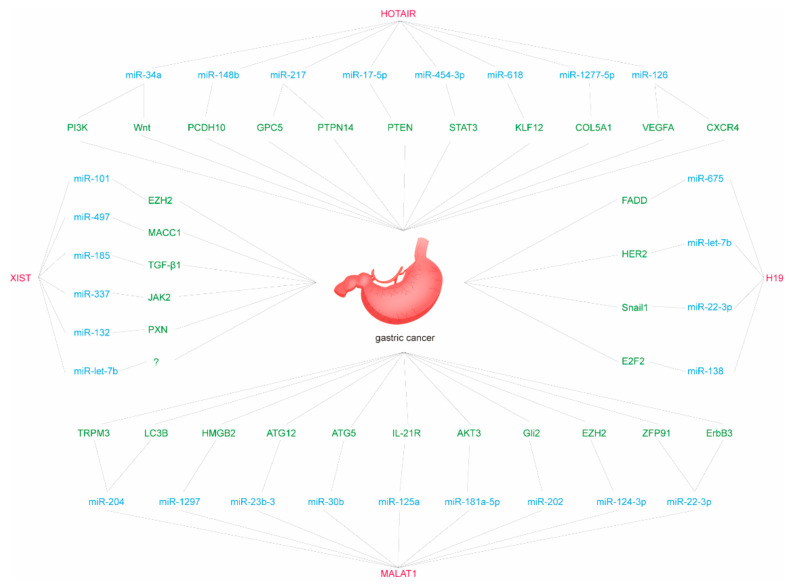
Representative lncRNA-mediated ceRNETs in GC.

In summary, some regulatory axes have been identified in the representative lncRNA-mediated ceRNETs that affect multiple hallmarks of GC progression, including proliferation, invasion, apoptosis, and migration (Figure 1 and Table 2). Studies have found that during the epithelial to mesenchymal transition (EMT) of gastric cancer, LncRNAs can act as ceRNAs to directly regulate the expression of E-cadherin and also to participate in the regulation of the expression of EMT-inducing transcription factors (EMT-TF) [123]. Further, many other lncRNAs also play the role of ceRNAs in GC. We have summarized studies on the role of lncRNAs as ceRNAs in GC during the past five years in Table 2.

## 5. circRNAs as ceRNAs in GC

circRNAs are closed loops in the cytoplasm, with neither a 5′cap structure nor a 3′polyadenylic acid tail structure. They were found in viroids for the first time [3]. With the development of RNA sequencing technology and in-depth research, it was found that circRNAs are widely transcribed in eukaryotes [165,166,167,168]. Compared with other linear ncRNAs, they have a high degree of conservation and stability. According to its components, they can be divided into three categories: exon circular RNAs (ecircRNAs) [169], intron circular RNAs (ciRNAs) [170], and exon–intron circular RNAs (EIciRNAs) [171], each of which has different molecular structures but have similar binding sites and regulatory functions, and provides a template for biosynthesis.

**Figure 2:**
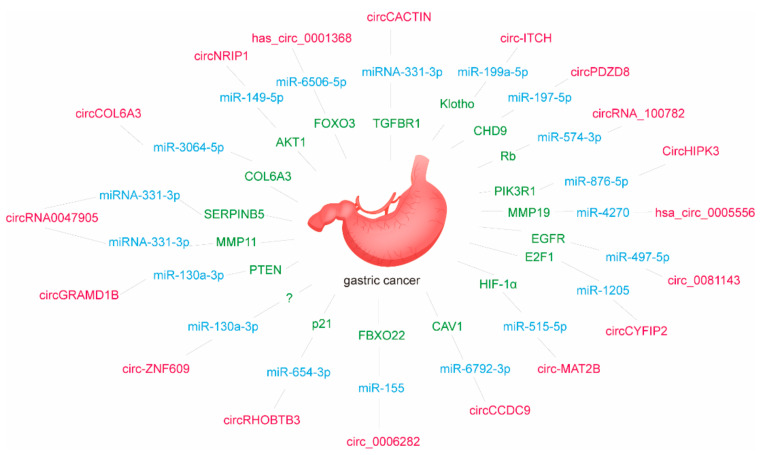
CircRNA-mediated ceRNETs in GC.

In recent years, there have been more studies on the function of circular RNAs as ceRNAs in GC. In 2017, researchers found that the expression of *circNRIP1* can up-regulate the AKT1 levels in GC cells and promote cell proliferation, migration, and invasion. Up-regulation of miR-149-5p can prevent the malignant behavior caused by *circNRIP1*. The *circNRIP1*/miR-149-5p/AKT1/mTOR axis is responsible for changes in GC cells’ metabolism and promotes the development of GC [172]. In 2019, researchers discovered a new type of circRNA, *has_circ_0001368*. The low expression of *has_circ_0001368* can promote tumor growth, and it plays a tumor suppressor effect in GC through the miR-6506-5p/FOXO3 axis [173]. In the same year, it was found that the expression of *circCOL6A3* and miR-3064-5p are inversely proportional. Overexpression of *circCOL6A3* promotes GC cell proliferation, migration, and apoptosis by eliminating the inhibitory effect on *COL6A3* induced by miR-3064-5p [174]. Studies have found that *circRNA0047905* can bind miR4516 and miR1227-5p, thereby reducing the inhibition of SERPINB5 and MMP11, activating the Akt/CREB signaling pathway, and promoting the progression of GC. *Circular RNA 0047905* may act as a tumor promoter in the pathogenesis of GC [175]. TGFBR1 is the receptor of the TGF-β ligand. Studies have found that *circCACTIN* promotes the progression of GC by sponging miRNA-331-3p and regulating the expression of TGFBR1 mRNA [176]. In studies to confirm the function of *circGRAMD1B*, it was found that *circGRAMD1B* inhibited the proliferation, migration, and invasion of GC cells by regulating miR-130a-3p-PTEN/p21 [177]. Through bioinformatics methods, it was found that miRNA-145-5p is the target gene of *circ-ZNF609*. Down-regulating the expression of miRNA-145-5p can partially reverse the effect of *circ-ZNF609* on the growth and migration of GC cells [178]. In 2020, researchers found that the expression of *circRHOBTB3* is low in GC tissues and cell lines. *circRHOBTB3* acts as a ceRNA for miR-654-3p and activates the p21 signaling pathway to inhibit GC’s growth. *circRHOBTB3* is promising as a new diagnostic marker, and therapeutic target for GC [179]. *circ_0006282* is a newly identified human circular RNA. Studies have found that its high expression can down-regulate miR-155, thereby activating the expression of FBXO22 and promoting the proliferation and migration of GC cells [180]. Similar to the expression of *circRHOBTB3*, *circCCDC9* was significantly down-regulated in GC tissues and cell lines. *circCCDC9* can inhibit tumor progression through the miR-6792-3p/CAV1 axis [181]. *circ-MAT2B* is mainly located in the cytoplasm and can act as a ceRNA to compete with miR-515-5p and increase the expression of HIF-1α [182]. *circCYFIP2* is significantly up-regulated in GC tissues. Research suggests that *circCYFIP2* may act as a carcinogenic circRNA to promote GC progression through the miR-1205/E2F1 axis [183]. *circ_0081143* modulates the abundance of miR-497-5p by making the miR-497-5p sponge. miR-497-5p directly targets EGFR and down-regulates *circ_0081143* to affect hypoxia-induced migration, invasion, and EMT of GC cells [184]. *circHIPK3* is derived from the homology domain-interacting protein kinase 3 (HIPK3) gene. In GC tissues and cell lines, *circHIPK3* is up-regulated. It regulates the miR-876-5p/PIK3R1 axis through the mechanism of ceRNA and mediates the proliferation, migration, and invasion of GC cells [185]. *circRNA_100782* is lowly expressed in GC. Studies have found that it can be used as a molecular sponge. It can bind to miR-574-3p to regulate the expression of the tumor suppressor gene Rb. This mechanism is closely related to the proliferation and invasion of GC [186]. In the study of *hsa_circ_0005556*, it was found that down-regulating the expression of *hsa_circ_0005556* can inhibit the growth of GC. The *hsa_circ_0005556*/miR-4270/MMP19 axis participates in the proliferation, migration, and invasion of GC cells through the ceRNA mechanism [187]. When *circPDZD8* is highly expressed, the survival rate of GC patients is poor. *circPDZD8* can up-regulate the expression of CHD9 by stimulating miR-197-5p to promote the proliferation and metastasis of GC [188]. The latest research shows that the expression level of *circ-ITCH* and miR-199-5p are negatively correlated in GC tissues. *circ-ITCH* can inhibit GC metastasis by acting as a sponge of miR-199a-5p and increasing Klotho expression [189]. So far, there are 18 miRNAs that have been identified as ceRNAs in the circRNA-mediated ceRNETs that affect multiple hallmarks of gastric progression, including proliferation, migration, invasion, and apoptosis (Figure 2 and Table 3).

## 6. Pseudogenes as ceRNAs in GC

Pseudogenes were once considered to be genomic fossils without bodily functions resulting from the accumulation of natural mutations of genes during biological evolution. Later, it was discovered that pseudogenes play a crucial role in gene transcription [190]. They can be used as ceRNAs to regulate gene transcription. In addition, pseudogenes can also regulate gene expression by interacting with RNA-binding proteins [191,192,193].

There are few studies on pseudogenes as ceRNAs in GC. In 2015, researchers reported for the first time that the pseudogene *FER1L4* acts as a ceRNA in the proliferation of GC. Down-regulation of FER1L4 increased the abundance of miR-106a-5p, decreased PTEN mRNA and protein quantity, and promoted GC proliferation [194]. In 2017, a study found that the pseudogene *PTENP1* of PTEN can be used as a ceRNA to regulate the expression of PTEN together with miR-106b/miR-93 [195]. The up-regulated expression of *PTENP1* can inhibit the proliferation, metastasis, and invasion of GC cells. In the latest study, it was found that *GBAP1* can competitively bind to miR-212-3p, promote GBA expression, and participate in GC development [196].

## 7. Conclusions

In summary, GC is a common gastrointestinal cancer with an insidious onset, and patients are often in the middle or late stage when they are diagnosed. It is important to understand the molecular mechanism of GC and to explore effective detection and treatment strategies.

The role of ncRNAs in tumors has been a hot spot in oncology research recently. The miRNA mechanism in tumors is now relatively clear, and lncRNAs, circRNAs, and pseudogenes have entered people’s fields of vision. Evidence shows that ceRNAs play an important regulatory role in GC. So far, researchers have established some RNA–miRNA–mRNA regulatory axes [197,198,199,200]. With the effective use of advanced bioinformatics tools, researchers can systematically construct more regulatory networks, and the identification of GC-related ceRNA networks should become more efficient and accurate. Some lncRNAs, circRNAs, and pseudogenes are found to act as ceRNAs. Studies showed that lncRNAs, circRNAs, and pseudogenes could promote the occurrence and development of tumors, inhibit tumor progression and metastasis, and regulate the sensitivity of tumor cells to chemotherapeutic drugs. However, the database of lncRNAs, circRNAs, and pseudogenes is not yet perfect.

Because studies usually use transfected oligonucleotides or expression vectors, there is a risk that the transfected oligonucleotide inhibitors (antagomir and miRNA sponge) may be collected by lysosomes and cannot cause miRNA activity. It is difficult to directly measure the potential activity of the introduced miRNAs. The current verification experiments are usually untested at the physiological level, artificially providing high quantification after the whole cell is lysed. Thus, the technologies to verify the effect of ceRNAs on target genes at the protein and RNA levels require a rigorous evaluation and should be complemented by studies in animal models to discover additional genes involved in cancer.

Moreover, the map of the complex lncRNA, circRNA, and pseudogene regulatory networks needs to be further improved and supplemented. However, researchers have mainly focused on a single axis or a single binding partner, and there is no uniform naming principle for lncRNAs, circRNAs, and pseudogenes. The secondary and indirect interactions may also affect the occurrence and development of GC and drug resistance. Therefore, further research should also pay attention to the complex lncRNA, circRNA, pseudogene, miRNA, and mRNA networks. Analyzing the lncRNA-specific molecular mechanisms underlying their biological function and transforming basic research into clinical application is still an enormous challenge.

## Figures and Tables

**Table 1:** Databases and resources for ceRNAs.

Tool Name	Functions	Website	Reference
ceRDB	Predict ceRNAs for specific mRNAs targeted by miRNAs by examining the co-occurrence of miRNA response elements in the mRNAs on a genome-wide basis.	http://www.oncomir.umn.edu/cefinder/ (accessed on 20 May 2021)	[53]
Linc2GO	MicroRNA–mRNA and microRNA–lincRNA interaction data were integrated to generate lincRNA functional annotations based on the ‘competing endogenous RNA hypothesis’.	http://www.bioinfo.tsinghua.edu.cn/~liuke/Linc2GO/index.html (not available on 20 May 2021)	[54]
StarBase v2.0	Provide the CLIP-Seq experimentally supported miRNA-mRNA and miRNA-lncRNA interaction networks to date.	http://starbase.sysu.edu.cn/ (accessed on 20 May 2021)	[55]
lnCeDB	A database of human lncRNAs (from GENCODE 19 version) that can potentially act as ceRNAs.	http://gyanxet-beta.com/lncedb (not available on 20 May 2021)	[56]
HumanViCe	Provide the potential ceRNA networks in virus-infected human cells.	http://gyanxet-beta.com/humanvice (not available on 20 May 2021)	[57]
Cupid	A method for simultaneous prediction of microRNA-target interactions and their mediated competitive endogenous RNA (ceRNA) interactions.	http://cupidtool.sourceforge.net/. (accessed on 20 May 2021)	[58]
miRSponge	Provide an experimentally supported resource for miRNA–sponge interactions and ceRNA relationships.	http://www.bio-bigdata.net/miRSponge. (not available on 20 May 2021)	[59]
SomamiR 2.0	A database of cancer somatic mutations in miRNA and their target sites that potentially alter the interactions between miRNAs and ceRNA including mRNAs, circRNA, and lncRNA.	http://compbio.uthsc.edu/SomamiR (accessed on 20 May 2021)	[60]
dreamBase	Provide insights into the transcriptional regulation, expression, functions, and mechanisms of pseudogenes as well as their roles in biological processes and diseases.	http://rna.sysu.edu.cn/dreamBase (accessed on 20 May 2021)	[61]
LncCeRBase	Encompasse 432 lncRNA–miRNA–mRNA interactions.	http://www.insect-genome.com/LncCeRBase (accessed on 20 May 2021)	[62]
LncACTdb 2.0	Provide comprehensive information of competing endogenous RNAs (ceRNAs) in different species and diseases.	http://www.bio-bigdata.net/LncACTdb/ (not available on 20 May 2021)	[63]
DIANA-LncBase v3.0	Provide correlations of miRNA–lncRNA pairs, as well as lncRNA expression profiles in a wide range of cell types and tissues.	www.microrna.gr/LncBase (accessed on 20 May 2021)	[64]
LnCeVar	Provide genomic variations that disturb lncRNA-associated ceRNA network regulation curated from the published literature and high-throughput data sets.	http://www.bio-bigdata.net/LnCeVar/ (not available on 20 May 2021)	[65]
ExoceRNA atlas	A repository of ceRNAs in blood exosomes.	https://www.exocerna-atlas.com/exoceRNA#/ (accessed on 20 May 2021)	[66]
Cerina	Predict biological functions of circRNAs based on the ceRNA model.	https://www.bswhealth.med/research/Pages/biostat-software.aspx. (accessed on 20 May 2021)	[67]
LnCeCell	Document cellular-specific lncRNA-associated ceRNA networks for personalised characterisation of diseases based on the ‘One Cell, One World’ theory.	http://www.bio-bigdata.hrbmu.edu.cn/LnCeCell/ (accessed on 20 May 2021)	[68]

**Table 2:** The mechanism of lncRNAs as ceRNAs in GC.

LncRNA	The Mechanism of ceRNA	Biological Functions	Reference
*BC032469*	miR-1207-5p/hTERT	Proliferation	[124]
*COL1A1-014*	miR-1273h-5p/CXCL12/CXCR4	Proliferation	[125]
*CRAL*	miR-505/CYLD/AKT	Resistance	[126]
*CTC-497E21.4*	miR-22/NET1	Proliferation, invasion	[127]
*DLX6-AS1*	miR-204-5p/OCT1	Proliferation, migration, invasion	[128]
*FLVCR1-AS1*	miR-155/c-Myc	Proliferation, invasion	[129]
*GAS5*	miR-23a/MT2A	Apoptosis	[130]
*H19*	miR-675/FADD/caspase 8/caspase 3	Proliferation	[102]
*H19*	miR-let-7c/HER2	Proliferation	[103]
*H19*	miR-22-3p/Snail1	Proliferation, migration	[105]
*H19*	miR-138/E2F2	Proliferation, invasion	[107]
*HNF1A-AS1*	miR-661/CDC34	Proliferation	[131]
*HOTAIR*	miR-126/VEGFA/PIK3R2	Resistance	[82]
*HOTAIR*	miR-34a/PI3K/Akt	Resistance	[83]
*HOTAIR*	miR-34a/Wnt/β-catenin	Resistance	[83]
*HOTAIR*	miR-217/GPC5 and PTPN14	Resistance	[84]
*HOTAIR*	miR-17-5p/PTEN	Proliferation	[85]
*HOTAIR*	miR-454-3p/STAT3/cyclin D1	Proliferation	[86]
*HOTAIR*	miR-126/CXCR4	Proliferation, migration	[87]
*HOTAIR*	miR-618/KLF12	Proliferation	[88]
*HOTAIR*	miR-1277-5p/COL5A1	Proliferation	[89]
*HOTAIR*	miR-148b/PCDH10	Proliferation	[91]
*IGF2-AS*	miR-503/SHOX2	Migration	[132]
*IGFL2-AS1*	miR-802/ARPP19	Proliferation, migration	[133]
*KCNQ1OT1*	microRNA-9-LMX1A	Proliferation, migration, invasion	[134]
*KCNQ1OT1*	miR-4319/DRAM2	Proliferation	[135]
*LINC00565*	miR-665/AKT3	Proliferation	[136]
*LINC01234*	miR-204-5p/CBFB	Proliferation	[137]
*LINC01606*	miR-423-5p/Wnt/β-catenin	Migration, invasion	[138]
*LINC01939*	miR-17-5p/EGR2	Migration	[139]
*LINC02163*	miR-593-3p/FOXK1	Proliferation	[140]
*LINC02532*	miR-129-5p and miR-490-5p	Proliferation, migration, invasion	[141]
*Lnc-ATB*	MiR-141-3p/TGFβ2	Proliferation	[142]
*lncR-D63785*	miR-422a/MEF2D	Chemotherapy sensitivity	[143]
*LOXL1-AS1*	miR-708-5p/USF1	Proliferation, migration	[144]
*LOXL1-AS1*	miR-142-5p/PIK3CA	Proliferation, migration	[145]
*MALAT1*	miR-202/Gli2	Proliferation	[114]
*MALAT1*	miR-1297/HMGB2	Proliferation, invasion	[115]
*MALAT1*	miR-23b-3/ATG12	Resistance	[116]
*MALAT1*	miR-30b/ATG5	Resistance	[117]
*MALAT1*	miR-125a/IL-21R	Proliferation, invasion	[118]
*MALAT1*	miR-181a-5p/AKT3	Proliferation	[119]
*MALAT1*	miR-204/LC3B	Proliferation	[120]
*MALAT1*	miR-204/transient receptor potential melastatin 3	Proliferation	[120]
*MALAT1*	miR-22-3p/ErbB3	Proliferation	[121]
*MALAT1*	miR-22-3p/ZFP91	Resistance	[121]
*MALAT1*	miR-124-3p/EZH2	Proliferation	[122]
*MYOSLID*	miR-29c-3p/MCL-1	Proliferation, inhibits apoptosis	[146]
*NORAD*	miR-608/FOXO6	Proliferation	[147]
*NORAD*	miR-214/Akt/mTOR	Proliferation, inhibits apoptosis	[148]
*NORAD*	miR-433-3p/ATG5,ATG12	Resistance	[149]
*PWRN1*	miR-425-5p/PTEN	Proliferation	[150]
*SLC25A5-AS1*	miR-19a-3p/PTEN/PI3K/AKT	Proliferation	[151]
*SNHG5*	miR-32/KLF4	Migration	[152]
*SPRY4-IT1*	miR-101-3p/AMPK	Proliferation, migration	[153]
*TINCR*	miR-375/PDK1	Proliferation	[154]
*TP73-AS1*	miR-194-5p/SDAD1	Proliferation, migration,	[155]
*TUBA4B*	miR-214 and miR-216a/b/PTEN	Proliferation, invasion	[156]
*UCA1*	miR-590-3p/CREB1	Proliferation, invasion	[157]
*UCA1*	miR-7-5p/EGFR	Migration	[158]
*UCA1*	miR-495-3p/SATB1	proliferation and invasion	[159]
*UCA1*	miR-203/ZEB2	Metastasis	[160]
*UCA1*	miR-26a/b, miR-193a, miR-214/PDL1	Proliferation, migration, immune escape and inhibits apoptosis	[161]
*UCA1*	miR-495/PRL-3	Proliferation, migration, invasion	[162]
*UCA1*	miR-513-3p/CYP1B1	Resistance	[163]
*XIST*	miR-101/EZH2	Proliferation, migration	[94]
*XIST*	miR-497/MACC1	Proliferation, invasion	[95]
*XIST*	miR-185/TGF-β1	Growth, migration and invasion	[96]
*XIST*	miR-337/JAK2	Proliferation, migration	[97]
*XIST*	miR-132/PXN	Proliferation, migration	[164]
*XIST*	XIST/miR-let-7b	Resistance	[98]

**Table 3:** The mechanism of circRNAs as ceRNAs in GC.

CircRNA	The Mechanism of ceRNA	Biological Functions	Reference
*circNRIP1*	miR-149-5p/AKT1/mTOR	Proliferation, migration, invasion	[172]
*circRNA has_circ_0001368*	miR-6506-5p/FOXO3	Proliferation	[173]
*circCOL6A3*	miR-3064-5p/COL6A3	Proliferation, migration, apoptosis	[174]
*circRNA0047905*	miR-4516/miR-1227-5p/SERPINB5/MMP11	Proliferation	[175]
*circCACTIN*	miRNA-331-3p/TGFBR1	Proliferation	[176]
*circGRAMD1B*	miR-130a-3p/PTEN/p21	Proliferation, migration, invasion	[177]
*circ-ZNF609*	miRNA-145-5p	Proliferation, migration	[178]
*circRHOBTB3*	miR-654-3p/p21	Proliferation	[179]
*circ_0006282*	miR-155/FBXO22	Proliferation, migration	[180]
*circCCDC9*	miR-6792-3p/CAV1	Proliferation	[181]
*circ-MAT2B*	miR-515-5p/HIF-1α	Proliferation	[182]
*circCYFIP2*	miR-1205/E2F1	Proliferation, invasion	[183]
*circ_0081143*	miR-497-5p/EGFR	migration, invasion, EMT	[184]
*CircHIPK3*	miR-876-5p/PIK3R1	Proliferation, migration, invasion	[185]
*circRNA_100782*	miR-574-3p/Rb	Proliferation, invasion	[186]
*hsa_circ_0005556*	miR-4270/MMP19	Proliferation, migration, invasion	[187]
*circPDZD8*	miR-197-5p/CHD9	Proliferation, migration	[188]
*circ-ITCH*	miR-199a-5p/Klotho	Migration	[189]

## Data Availability

No data are available for this review article.

## Abbreviations

AKT	AKT serine/threotine kinase 1
AKT3	AKT serine/threonine kinase 3
AMPK	Protein kinase AMP-activated catalytic subunit alpha 1
ARPP19	CAMP-regulated phosphoprotein 19
ATG5	Autophagy-related 5
ATG12	Autophagy-related 12
CAV1	Caveolin 1
CBFB	Core-binding factor subunit beta
CDC34	Cell division cycle 34, ubiqiutin-conjugating enzyme
ceRNAs	Competitive endogenous RNAs
ceRNETs	ceRNA networks
CHD9	Chromodomain helicase DNA-binding protein 9
ciRNAs	Intron circular RNAs
COL1A2	Collagen type I alpha 2 chain
COL3A1	Collagen type III alpha 1 chain
COL4A1	Collagen type IV alpha 1 chain
COL5A1	Collagen type V alpha 1 chain
COL5A2	Collagen type V alpha 2 chain
CREB1	CAMP-responsive element-binding protein 1
CXCL12	C-X-C motif chemokine ligand 12
CXCR4	C-X-C motif chemokine receptor 4
CYLD	CYLD lysine 63 deubiquitinase
CYP1B1	Cytochrome P450 family 1 subfamily B member 1
DRAM2	DNA-damage-regulated autophagy modulator 2
E2F1	E2F transcription factor 1
E2F2	E2F transcription factor 2
ecircRNAs	Exon circular RNAs
EGFR	epidermal growth factor receptor
EGR2	Early growth response 2
EIciRNAs	Exon–intron circular RNAs
EMT	Epithelial to mesenchymal transition
EMT-TF	EMT-inducing transcription factor
ErbB3	Erb-B2-receptor tyrosine kinase 3
EZH2	Enhancer of zeste 2 polycomb-repressive complex 2 subunit
FADD	Fas-associated via death domain
FBXO22	F-box protein 22
FOXK1	Forkhead box K1
FOXO3	Forkhead box O3
FOXO6	Forkhead box O6
GBA	Glucosylceramidase beta
GC	Gastric cancer
GEO	Gene Expression Omnibus microarray datasets
Gli2	GLI family zinc finger 2
GPC5	Glypican-5
H19	H19-imprinted maternally-expressed transcript
HER2	Erb-B2-receptor tyrosine kinase 2
HIF-1α	Hypoxia-inducible factor 1 subunit alpha
HIPK3	Homeodomain-interacting protein kinase 3
HMGB2	High-mobility group box 2
HOTAIR	HOX transcript antisense RNA
hTERT	Human telomerase reverse transcriptase
IGF2	Insulin-like growth factor 2
IL-21R	Interleukin 21 Receptor
JAK2	Janus kinase 2
KLF4	Kruppel-like Factor 4
LC3B	Microtubule-associated protein 1 light chain 3 beta
LMX1A	LIM homeobox transcription factor 1 alpha
lncRNAs	Long non-coding RNAs
MACC1	MET transcriptional regulator MACC1
MALAT1	Metastasis-associated lung adenocarcinoma transcript 1
MCL-1	MCL1 apoptosis regulator, BCL2 family member
MEF2D	Myocyte enhancer factor 2D
miRNA–RISC	miRNA-mediated silencing complex
miRNAs	MicroRNAs
MMP11	Matrix metallopeptidase 11
MMP19	Matrix metallopeptidase 19
MRE	miRNA response element
mRNAs	Messenger RNAs
MT2A	Metallothionein 2A
mTOR	Mechanistic target of rapamycin kinase
NET1	Neuroepithelial cell transforming 1
OCT1	POU class 2 homeobox 1
PIK3CA	Phosphatidylinositol-4,5-bisphosphate 3-kinase catalytic subunit alpha
PIK3R1	Phosphoinositide-3-kinase regulatory subunit 1
PIK3R2	Phosphoinositide-3-kinase regulatory subunit 2
PCDH	Protocadherin 10
PDK1	Pyruvate dehydrogenase kinase 1
PDL1	CD274 molecule
PRC2	Polycomb repressive complex 2
PRL-3	Protein tyrosine phosphatase 4A3
PTEN	Phosphatase and tensin homolog
PTPN14	Protein tyrosine phosphatase non-receptor type 14
PXN	Paxillin
SATB1	SATB homeobox 1
SDAD1	SDA1 domain-containing 1
SERPINB5	Serpin family B member 5
SHOX2	Short stature homeobox 2
Snail1	Snail family transcriptional repressor 1
STAT3	Signal transducer and activator of transcription 3
TCGA	Cancer Genome Atlas database
TGF-β1	Transforming growth factor beta 1
TGF-β2	Transforming growth factor beta 2
TGFBR1	Transforming growth factor beta receptor 1
USF1	Upstream transcription factor 1
VEGFA	Vascular endothelial growth factor A
XIST	X inactive specific transcript
ZEB2	Zinc finger E-box-binding homeobox 2
ZFP91	ZFP91 zinc finger protein, atypical E3 ubiquitin ligase
